# Self-learning adaptive neuro-fuzzy approximation of robust control behavior in electric power steering systems

**DOI:** 10.1371/journal.pone.0334539

**Published:** 2025-10-24

**Authors:** Tuan Anh Nguyen, Tran Minh Ngoc Do, Thi Thu Huong Tran, The Truc Nguyen, Quang Vinh Tran

**Affiliations:** 1 Faculty of Mechanical Engineering, Thuyloi University, Hanoi, Vietnam; 2 School of Mechanical Engineering, Hanoi University of Science and Technology, Hanoi, Vietnam; 3 University of Wisconsin-Whitewater, Whitewater, Wisconsin, Unites State of America; 4 Phenikaa school of Engineering, Phenikaa University, Hanoi, Vietnam; Instituto Politecnico Nacional, MEXICO

## Abstract

Data training algorithms based on Artificial Intelligence (AI) often encounter overfitting, underfitting, or bias issues. This article presents the design of a hybrid self-learning algorithm to address the above challenges. The proposed approach is developed by integrating fuzzy logic and neural network structures into an Adaptive Network-Based Fuzzy Inference System (ANFIS), which leverages the strengths of both components. This integration is considered a key contribution of the study. Compared to conventional training algorithms, the proposed ANFIS demonstrates high training accuracy while maintaining strong interpolation and prediction capabilities, even under varying conditions. The model is designed with three inputs and one output, trained using data derived from a high-performance robust controller for Electric Power Steering (EPS) systems. Simulation results show that the training error of the proposed ANFIS remains below 1.7% in well-trained cases and under 6.1% in interpolation scenarios. Moreover, the algorithm maintains a prediction error of less than 9.3% when applied to scenarios outside the training domain. The issue of overfitting is significantly resolved, unlike in the case of the Backpropagation Neural Network (BPNN), which is used as a benchmark for comparison. Overall, the proposed algorithm significantly improves data training accuracy and generalization performance.

## 1. Introduction

The Hydraulic Power Steering (HPS) system was invented in the 1950s and has a cumbersome and complex structure. It was not until the end of the 20th century that the Electric Power Steering (EPS) system was introduced to replace the traditional HPS. Today, the EPS system is equipped to handle most new car models [1]. EPS systems offer many advantages over traditional steering systems, including compact structure, high assisted performance, energy saving, and environmental friendliness [2]. This system can be easily integrated into many modern electric and self-driving vehicles.

Many studies on EPS control have been published in recent years. In [3], Turan designed an improved Proportional-Integral-Derivative (PID) algorithm to control the performance of an EPS system. The controller parameters were tuned through an optimization algorithm to minimize the total error, including maximum shoot and settling time errors. Hanifah et al. designed an Ant Colony Optimization (ACO) algorithm to tune the PID controller parameters to reduce the power consumption of the EPS system [4]. The results in [4] showed that the maximum assisted current is slightly reduced compared to the conventional PID controller. Zheng and Wei utilized a fuzzy algorithm to tune the PID control parameters [5]. They concluded that the motor current tracking performance is improved by 75.2% compared to Active Disturbance Rejection Control (ADRC), while the yaw rate overshoot is also reduced by more than 70%. Another application of Fuzzy Logic System (FLS) in tuning the parameters of PI controller was also introduced in [6] by Dai et al. A significant improvement in control performance was seen in [7] where Li et al. utilized Back Propagation Neural Network (BPNN) to self-tune the control parameters of PID controller based on the error between actual and desired current signals.

The PID algorithm fails to control systems formed with Multiple Inputs and Multiple Outputs (MIMO). In [8], Mehrabi et al. designed a Linear Quadratic Gaussian (LQG), which was a combination of Linear Quadratic Regulator (LQR) and a Kalman filter, to solve this problem. Simulation results in [8] showed that the performance of LQG in controlling the system is superior to that of conventional PID. Liu et al. designed a Genetic Algorithm (GA) to optimize LQR control, which was utilized to control the EPS system [9]. In [10], Nguyen used the FLS to adjust the inputs of Linear Quadratic Tracking (LQT) control to improve the tracking error. However, the influence of sensor noise was not considered. Yamamoto designed Linear Parameter-Varying (LPV) control based on an H_∞_/H_2_ PI observer to improve the performance of the EPS system. The results in [11] showed an average error of about 5.75%. However, the performance of conventional controllers, which were designed for linear systems, was not guaranteed under many different conditions. This was verified through simulation results presented in [12–14].

Several high-performance, robust control techniques have recently been developed and applied to control EPS systems. A controller based on the combination of Backstepping Control (BSC) and PID was introduced in [15] by Nguyen and Nguyen. The final control signal was optimized through the corresponding gain coefficients of the two component controllers. However, the theoretical stability of the system was not fully demonstrated. In [16], Nguyen and Iqbal utilized the GA to tune the parameters of the BSPID controller. The input of the BSC was the output of the PID control, instead of summing the two signals as mentioned in [15]. The simulation results in [16] showed that the tracking performance of the BSPID-GA control is significantly improved, while the traditional backstepping control suffers from phase shift. An innovation was seen in [17] where the BSC input was calibrated through an FLS formed based on 25 fuzzy rules. A combination of PID, FLS, and BSC for improving the system performance was presented in [18]. However, the error in the motor current was quite significant and needed to be addressed in further work.

The Sliding Mode Control (SMC) technique effectively controls automotive mechatronic systems. In [19], Marouf et al. designed an SMC loop algorithm integrated with an SM observer. The computational results in [19] showed that the errors in steering column angular acceleration and motor angular acceleration are pretty significant. An SMC mechanism with disturbance rejection was introduced in [20] by Khasawneh and Das. The results of [20] revealed that the influence of the chattering phenomenon is significant when utilizing the SMC technique to control the EPS system. In [21], Kim et al. conducted an experiment with MicroAutobox II and a drive-kit test to demonstrate the performance of the SMC mechanism, which was integrated with a disturbance observer. A combination of SMC and a Kalman filter was implemented in [22] by Li et al. In [23], Kim et al. designed an Extended State Observer (ESO), which was integrated with the SMC mechanism, to estimate the external disturbance. Overall, the results in [21–24] showed the negative impact of chattering, a phenomenon considered inherent in this control mechanism. A combination of SMC and PID was implemented in [25,26] to improve the system performance. However, the calculation of road torque was ignored, which was considered a significant limitation. In [27], Lu et al. designed a fuzzy SMC scheme to eliminate the impact of chattering. Nguyen introduced the design of an adaptive sliding surface based on the FLS to improve the performance of the SMC framework [28]. Some combinations of SMC and BSC were introduced in [29,30]. Overall, the impact of chattering was primarily but not completely resolved.

External disturbances cause some negative impacts on EPS systems. In [31], Na et al. designed the ADRC technique for steering wheel torque tracking. The structure of the control mechanism proposed in [31] consisted of a tracking differentiator, a Linear ESO (LESO), and a state feedback control law. The computational results showed that the errors of conventional differentiators are much larger than those of tracking differentiators. A study by Zheng and Wei verified that the tracking accuracy in assisted current is improved by 45.8% compared to conventional PID. In addition, the performance of the ADRC algorithm was superior to that of fuzzy PID under extreme conditions, as verified by the computational results in [32]. Recently, Nguyen and Nguyen developed a new control mechanism, which integrated SMC and nonlinear ADRC (NADRC) to improve tracking performance and eliminate the effects of chattering. The calculation results in [33] showed that the average tracking error is only about 2.0% when the vehicle is steering at 30 km/h and no more than 3.5% when steering at 70 km/h.

Several applications of intelligent control have been applied to control the performance of modern EPS systems. In [34], Alabe et al. utilized a deep learning solution to detect steering system anomalies by combining long short-term memory and an autoencoder. Hartono et al. designed a control strategy for the EPS system based on Artificial Neural Network (ANN) using a BPNN algorithm. This aimed to learn the system’s nonlinear dynamics [35]. In [36], Amirkhani et al. designed a radial basis function-based adaptive ANN to approximate nonlinear dynamics to improve the control performance of the EPS system. They utilized an optimal algorithm called Coronavirus Disease (COVID) to calculate the optimal parameters for the controller. An ANN mechanism based on Reinforced Gain (RG) was designed in [37] to approximate parameter uncertainties and disturbances. However, the tracking error was still significant. In [38], Lin et al. presented the Wavelet Fuzzy Neural Network (WFNN) design as an uncertainty estimator to overcome the shortcomings of the SMC framework. The control method proposed in [38] was utilized to control the performance of a permanent magnet synchronous motor, which was equipped on the EPS system as an actuator. Another application of WFNN was presented in [39] by Hung et al. for online training purposes to control the assisted motor. This control mechanism was formed by combining asymmetric membership functions and BPNN algorithms. An Adaptive Network-Based Fuzzy Inference System (ANFIS) was presented in [40] by Ramos-Fernández et al. for training control inputs obtained from a PD controller. The simulation results in [40] showed that the training error is significant, which is caused by two reasons: the input data is not highly accurate, and the structure of the ANFIS algorithm is unsuitable. Overall, intelligent self-learning algorithms (including BPNN, deep learning, machine learning, WFNN, ANFIS, and others) are highly effective in training data within a specific range. However, the tracking error may increase if the training data is insufficient or the algorithm structure is not optimally calculated.

### 1.1. Research gaps

Despite the considerable progress in developing control strategies for EPS systems, several research gaps remain. Firstly, conventional control approaches, such as LQR, LQT, PID, LPV, and lead-lag control, often fall short when applied to highly nonlinear and complex systems characteristic of EPS dynamics. Secondly, external disturbances continue to degrade tracking accuracy, even under robust control techniques such as BSC and ADRC. Thirdly, the chattering phenomenon inherent in SMC significantly affects system stability and actuator performance, especially under varying operating conditions.

Fourthly, using ANN-based algorithms presents challenges related to overfitting, underfitting, and bias, particularly when the training data is limited or not sufficiently representative of all operating scenarios. These factors can lead to poor generalization and increased tracking errors. Finally, designing and implementing a nonlinear, robust controller often requires prior knowledge of the system’s internal structure or control model, which may not be readily available or well-defined in practice.

These drawbacks highlight critical gaps for further investigation to improve EPS control systems’ performance, robustness, and generalizability.

### 1.2. Key contributions

This work is motivated by the research gaps identified in recent EPS control strategies. In this article, we propose designing an intelligent self-learning algorithm based on integrating the FLS and ANN, known as the Adaptive Neuro-Fuzzy Inference System (ANFIS). Unlike the approach in [40], the proposed ANFIS is constructed with a more advanced architecture to train large-scale datasets with inconsiderable error effectively.

Furthermore, the input data used for training is derived from a high-performance, robust control mechanism that combines nonlinear ADRC and SMC frameworks. The primary objective of employing ANFIS in this work is to develop a control mechanism capable of adapting to diverse operating conditions, even when the structure of the original control algorithm, which is used to generate the training data, is unclear.

To the best of the authors’ knowledge, applying ANFIS to train the control input for EPS systems remains largely unexplored, except for the preliminary work in [40]. The ANFIS algorithm demonstrates strong capabilities in data learning, thereby improving accuracy in inference, interpolation, and prediction tasks. Additionally, this solution provides significant advantages over traditional ANN-based methods by mitigating the effects of overfitting, underfitting, and bias in the training process.

Several successful applications of ANFIS have been introduced in other automotive areas, such as active suspension systems [41,42], anti-rollover control [43,44], and energy management systems in electric vehicles [45,46], which further offers its potential effectiveness in EPS control.

This article is organized as follows: the introduction section presents the literature review, research gaps, and key contributions. The following section describes the design of the proposed robust control scheme and the development of the proposed ANFIS algorithm. Simulation results and discussions are presented in the third section. Finally, the conclusion outlines potential directions for future work.

## 2. Mathematical model

This section presents the mathematical model design of the EPS system, control mechanism, and intelligent self-learning algorithm.

### 2.1. Electric power steering model

**Fig 1:**
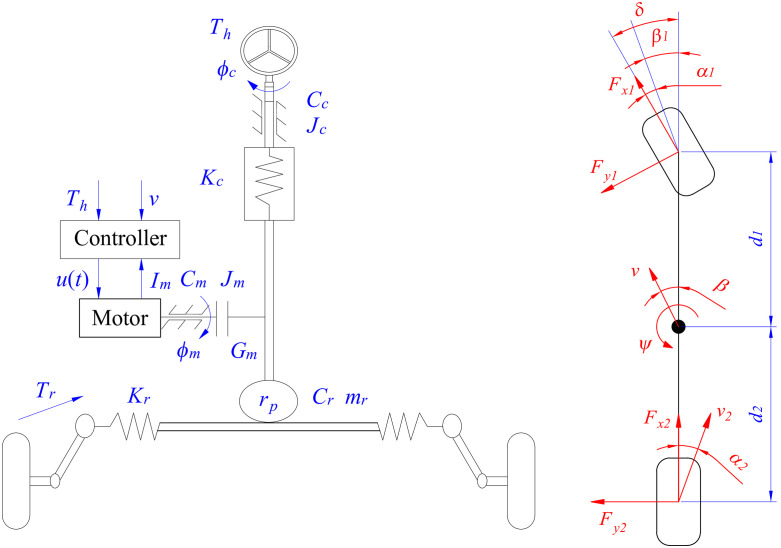
System models. (a) Column EPS system, (b) Vehicle dynamics system.

In this article, the training data used for the algorithm is derived from a robust controller designed for the EPS system. The structure of a Column Electric Power Steering (CEPS) system is illustrated in Fig 1a, which comprises a steering wheel, a rack-and-pinion mechanism, an actuator (steering motor) with a pair of gears, sensors, and an Electronic Control Module (ECM).

The system dynamics are introduced in Equations (1) and (2), based on D’Alembert’s principle.


$$J_{c}{\ddot{\phi }}_{c}+C_{c}{\dot{\phi }}_{c}+K_{c}\left(\phi_{c}-\frac{\phi_{m}}{G_{m}}\right)=T_{h}$$ (1)

$$\left(J_{m}+m_{r}\frac{r_{p}^{2}}{G_{m}^{2}}\right){\ddot{\phi }}_{m}+\left(C_{m}+C_{r}\frac{r_{p}^{2}}{G_{m}^{2}}\right){\dot{\phi }}_{m}+\frac{K_{c}+K_{r}r_{p}^{2}}{G_{m}^{2}}\phi_{m}=\frac{K_{c}}{G_{m}}\phi_{c}+K_{t}i_{m}-\frac{T_{r}}{G_{m}}$$ (2)

The symbols appearing in the above equations are explained as follows: *ϕ*_*c*_ is steering column angle, *J*_*c*_ is column inertia moment, *C*_*c*_ is column damping, *K*_*c*_ is column stiffness, *ϕ*_*m*_ is steering motor angle, *G*_*m*_ is steering motor ratio, *T*_*h*_ is hand torque, *J*_*m*_ is motor inertia moment, *r*_*p*_ is pinion radius, *m*_*r*_ is rack mass, *C*_*r*_ is rack damping, *C*_*m*_ is motor damping, *K*_*t*_ is assisted coefficient, *i*_*m*_ is steering motor current, and *T*_*r*_ is reaction torque.

The steering motor dynamics are represented by Equation (3), where *L*_*m*_ is the motor inductance, *r*_*m*_ is the motor resistance, and *u*(*t*) is the control signal.


$$K_{t}{\dot{\phi }}_{m}+L_{m}{\dot{I}}_{m}+r_{m}I_{m}=u\left(t\right)$$ (3)

Reaction torque is the resisting torque from the road surface, which consists of two components shown in (4), where *T*_*er*_ is external resistance (disturbance) and *T*_*ir*_ is internal resistance. Equation (5) represents an approximate method of calculating the internal resistance value, where *λ*_*kin*_ is the kingpin angle, *λ*_*cas*_ is the caster angle, *d*_*k*_ is the knuckle arm length, and *d*_*c*_ is the caster trail.


$$T_{r}=T_{er}+T_{ir}$$ (4)

$$T_{ir}\approx r_{p}d_{c}F_{y1}\frac{cos^{2}\left(\lambda_{kin}\right)cos^{2}\left(\lambda_{cas}\right)}{d_{k}}$$ (5)

A linear tire model calculates the value of lateral tire force (*F*_*y*_), which is presented in (6), where *K*_*α*_ is the tire cornering stiffness.


$$F_{yi}=-K_{\alpha i}\alpha_{i}$$ (6)

In the linear deformation region, the change in tire slip angle (*α*) is determined by Equation (7), where *δ* is the steering angle, *d* is the axle distance, *v*_*x*_ and *v*_*y*_ are the longitudinal and lateral speeds, respectively, and *ψ* is the yaw angle. The index *i* denotes the considered position (*i* = 1 for the front axle, and *i* = 2 for the rear axle).


$$\alpha_{i}=\frac{v_{y}+{\left(-1\right)}^{i\;+\;1}d_{i}\dot{\psi }}{v_{x}}-\delta_{i}$$ (7)

Longitudinal and lateral speed are represented by the heading angle, denoted by *β*, according to Equation (8).


$$tan\beta =\frac{v_{y}}{v_{x}}$$ (8)

The variation in yaw angle (*ψ*) is determined through a linear dynamic model (Fig 1b), which is described by Equations (9), (10), and (11). The symbols used in these equations include *m* (vehicle mass), *F*_*x*_ (longitudinal tire force), and *J*_*ψ*_ (yaw inertia moment).


$$m\left({\dot{v}}_{x}-\dot{\psi }v_{y}\right)=F_{x1}cos\delta +F_{x2}-F_{y1}\sin\delta$$ (9)

$$m\left({\dot{v}}_{y}+\dot{\psi }v_{x}\right)=F_{y1}cos\delta +F_{y2}+F_{x1}\sin\delta$$ (10)

$$J_{\psi }\ddot{\psi }=d_{1}\left(F_{x1}\sin\delta +F_{y2}cos\delta \right)-d_{2}F_{y2}$$ (11)

Assume that the steering angle is slight while the moving speed is constant. Equations (9) to (11) are rewritten according to (12).


$$\left[\begin{array}{c} {}*20c\dot{\beta }\\ \ddot{\psi } \end{array}\right]=-\sum_{i=1}^{2}\left[\begin{array}{cc} {}*20c\frac{K_{\alpha i}}{mv} & \frac{{\left(-1\right)}^{i+1}d_{i}K_{\alpha i}}{mv^{2}}+1\\ \frac{{\left(-1\right)}^{i+1}d_{i}K_{\alpha i}}{J_{\psi }} & \frac{d_{i}^{2}K_{\alpha i}}{J_{\psi }v} \end{array}\right]\left[\begin{array}{c} {}*20c\beta \\ \dot{\psi } \end{array}\right]+\left[\begin{array}{c} {}*20c\frac{K_{\alpha 1}}{mv}\\ \frac{d_{1}K_{\alpha 1}}{J_{\psi }} \end{array}\right]\left[\delta \right]$$ (12)

### 2.2. Ideal assisted torque map

**Fig 2:**
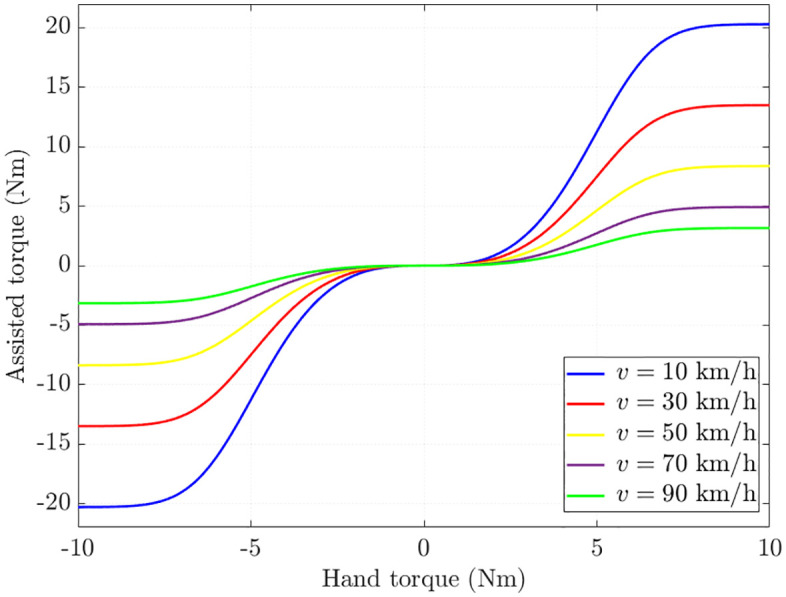
Ideal assisted map.

Designing an EPS controller aims to ensure the control performance adheres to a predefined assisted characteristic. Fig 2 illustrates the ideal assisted curves of the EPS system. In general, the assisted torque (*T*_*a*_) remains nearly zero when the hand torque (*T*_*h*_) is below a lower threshold (*T*_*h*_ < *T*_*h_min*_). Conversely, *T*_*a*_ approaches a saturation level when hand torque exceeds its upper limit (*T*_*h*_ > *T*_*h_max*_). Within the stable operating range (*T*_*h_min*_ ≤ *T*_*h*_ ≤ *T*_*h_max*_), the assisted torque varies nonlinearly as a function of both hand torque and vehicle speed. These smooth characteristic curves are introduced in Equation (13), where *k*_*g*_, *k*_*v*_, and *k*_*a*_ are curve coefficients, and *e* is the mathematical constant approximately 2.71828.

These are smooth and continuous curves described by the mathematical function, as opposed to the piecewise-defined curves presented in [1,5,7,10,14–18,28–30,32]. This approach improves steering comfort and driving smoothness while ensuring vehicle stability and safety.


$$T_{a}\left(v,T_{h}\right)=k_{g}\left(k_{v1}v^{2}+k_{v2}v+k_{v3}\right)\left(\frac{\text{1}}{1+e^{-k_{a}T_{h}^{3}}}-\frac{\text{1}}{1+e^{k_{a}T_{h}^{3}}}\right)$$ (13)

### 2.3. Robust control design

As presented above, the training data in this article is referenced from a robust nonlinear controller combining NADRC and SMC techniques. The control design process is referenced in [33] and briefly described below.

The state variables are arranged in the order they appear in Equation (14).


$$\left[\begin{array}{ccccc} {}*20cx_{1} & x_{2} & x_{3} & x_{4} & x_{5} \end{array}\right]=\left[\begin{array}{ccccc} {}*20c\phi_{c} & {\dot{\phi }}_{c} & \phi_{m} & {\dot{\phi }}_{m} & I_{m} \end{array}\right]$$ (14)

Taking the derivatives of the state variables (*x*_*i*_) in turn, we get Equations (15) to (19).


$${\dot{x}}_{1}=x_{2}$$ (15)

$${\dot{x}}_{2}=-\frac{K_{c}}{J_{c}}x_{1}-\frac{C_{c}}{J_{c}}x_{2}+\frac{K_{c}}{J_{c}G_{m}}x_{3}+\frac{T_{h}}{J_{c}}$$ (16)

$${\dot{x}}_{3}=x_{4}$$ (17)

$${\dot{x}}_{4}=\frac{K_{c}G_{m}}{J_{m}G_{m}^{2}+m_{r}r_{p}^{2}}x_{1}-\frac{K_{c}+K_{r}r_{p}^{2}}{J_{m}G_{m}^{2}+m_{r}r_{p}^{2}}x_{3}-\frac{C_{m}G_{m}^{2}+C_{r}r_{p}^{2}}{J_{m}G_{m}^{2}+m_{r}r_{p}^{2}}x_{4}+\frac{K_{t}G_{m}^{2}}{J_{m}G_{m}^{2}+m_{r}r_{p}^{2}}x_{5}-\frac{G_{m}T_{r}}{J_{m}G_{m}^{2}+m_{r}r_{p}^{2}}$$ (18)

$${\dot{x}}_{5}=-\frac{K_{t}}{L_{m}}x_{4}-\frac{r_{m}}{L_{m}}x_{5}+\frac{\text{1}}{L_{m}}u\left(t\right)$$ (19)

The Nonlinear Extended State Observer (NESO) is established to estimate state variable variations, as Nguyen and Nguyen proposed in [33]. Only the steering column angle is assumed to be directly measured by a physical sensor, while the remaining states are estimated through the nonlinear observer. Let *e*_*ε*_ denote the estimation error between the observed signal and the sensor measurement, as defined in Equation (20). The resistant torque (*T*_*r*_) is considered a disturbance and is estimated via an augmented state variable, as shown in Equation (21).


$$e_{\varepsilon }=x_{1}-{\hat{x}}_{1}$$ (20)

$${\hat{x}}_{6}=T_{r}$$ (21)

The structure of the proposed NESO is presented in Equations (22) to (27).


$${\dot{\hat{x}}}_{1}={\hat{x}}_{2}+\varepsilon_{1}$$ (22)

$${\dot{\hat{x}}}_{2}=-\frac{K_{c}}{J_{c}}{\hat{x}}_{1}-\frac{C_{c}}{J_{c}}{\hat{x}}_{2}+\frac{K_{c}}{J_{c}G_{m}}{\hat{x}}_{3}+\frac{T_{h}}{J_{c}}+\varepsilon_{2}$$ (23)

$${\dot{\hat{x}}}_{3}={\hat{x}}_{4}+\varepsilon_{3}$$ (24)

$$\begin{array}{c} {\dot{\hat{x}}}_{4}=\frac{K_{c}G_{m}}{J_{m}G_{m}^{2}+m_{r}r_{p}^{2}}{\hat{x}}_{1}-\frac{K_{c}+K_{r}r_{p}^{2}}{J_{m}G_{m}^{2}+m_{r}r_{p}^{2}}{\hat{x}}_{3}-\frac{C_{m}G_{m}^{2}+C_{r}r_{p}^{2}}{J_{m}G_{m}^{2}+m_{r}r_{p}^{2}}{\hat{x}}_{4}\\ +\frac{K_{t}G_{m}^{2}}{J_{m}G_{m}^{2}+m_{r}r_{p}^{2}}{\hat{x}}_{5}-\frac{G_{m}T_{r}}{J_{m}G_{m}^{2}+m_{r}r_{p}^{2}}+\varepsilon_{4} \end{array}$$ (25)

$${\dot{\hat{x}}}_{5}=-\frac{K_{t}}{L_{m}}{\hat{x}}_{4}-\frac{r_{m}}{L_{m}}{\hat{x}}_{5}+\frac{\text{1}}{L_{m}}u\left(t\right)+\varepsilon_{5}$$ (26)

$${\dot{\hat{x}}}_{6}=\varepsilon_{6}$$ (27)

Observed gains (*ε*) are determined by a nonlinear model, which is introduced in (28) and (29), where *g*_*i*_ are observed coefficients, *β*_*i*_ are threshold parameters, and *φ*_*i*_ are tuning parameters.


$$\varepsilon_{i}=g_{i}fal\left(e_{\varepsilon };\varphi_{i};\beta_{i}\right)$$ (28)

$$fal\left(e_{\varepsilon };\varphi_{i};\beta_{i}\right)=\{\begin{array}{cc} {}*20c\frac{e_{\varepsilon }}{\beta_{i}^{1-\varphi_{i}}} & \left|e_{\varepsilon }\right|\leq \beta_{i}\\ sign\left(e_{\varepsilon }\right){\left|e_{\varepsilon }\right|}^{\varphi_{i}} & \left|e_{\varepsilon }\right|>\beta_{i} \end{array}$$ (29)

This work uses a Nonlinear Tracking Differentiator (NTD) to remove the effects of disturbance and smooth the reference signal. The mathematical model of NTD is presented in Equations (30) and (31), where *y*_*i*_ are updated outputs and *k*_*y*_ are positive constants.


$${\dot{y}}_{1}=y_{2}$$ (30)

$${\dot{y}}_{2}=-k_{y1}e_{y1}-k_{y2}sat\left(e_{y2}\right)+{\ddot{x}}_{3\_ref}=y_{3}$$ (31)

The errors of the NTD are denoted as *e*_*y*1_ and *e*_*y*2_ and are expressed in (32) and (33), respectively.


$$e_{y1}=y_{1}-x_{3\_ref}$$ (32)

$$e_{y2}={\dot{e}}_{y1}=y_{2}-{\dot{x}}_{3\_ref}$$ (33)

Substituting Equation (31) into (33), we get (34).


$${\dot{e}}_{y2}=-k_{y1}e_{y1}-k_{y2}sat\left(e_{y2}\right)$$ (34)

Let the error between the observed and smoothed signals be *e*_3_, illustrated in (35).


$$e_{3}=y_{1}-{\hat{x}}_{3}$$ (35)

Taking the derivative of *e*_3_ twice, we obtain Equations (36) and (37), respectively.


$${\dot{e}}_{3}={\dot{y}}_{1}-{\dot{\hat{x}}}_{3}=y_{2}-{\hat{x}}_{4}-\varepsilon_{3}$$ (36)

$${\ddot{e}}_{3}={\dot{y}}_{2}-{\dot{\hat{x}}}_{4}-{\dot{\varepsilon }}_{3}=y_{3}-\sum_{i=1}^{6}b_{i}{\hat{x}}_{i}-\varepsilon_{4}-{\dot{\varepsilon }}_{3}$$ (37)

The *b*_*i*_ symbols are explained according to (38) to (43).


$$b_{1}=\frac{K_{c}G_{m}}{J_{m}G_{m}^{2}+m_{r}r_{p}^{2}}$$ (38)

$$b_{2}=0$$ (39)

$$b_{3}=-\frac{K_{c}+K_{r}r_{p}^{2}}{J_{m}G_{m}^{2}+m_{r}r_{p}^{2}}$$ (40)

$$b_{4}=-\frac{C_{m}G_{m}^{2}+C_{r}r_{p}^{2}}{J_{m}G_{m}^{2}+m_{r}r_{p}^{2}}$$ (41)

$$b_{5}=\frac{K_{t}G_{m}^{2}}{J_{m}G_{m}^{2}+m_{r}r_{p}^{2}}$$ (42)

$$b_{6}=-\frac{G_{m}}{J_{m}G_{m}^{2}+m_{r}r_{p}^{2}}$$ (43)

Although the sensor directly measures the steering column angle (*x*_1_), it is not the control target in this study. Instead, the controlled variable is the steering motor angle, denoted by *z* as defined in Equation (44).


$$z={\hat{x}}_{3}$$ (44)

Taking the third derivative of *z*, we get (45), (46), and (47), where *d* is the estimated total disturbance.


$$\dot{z}={\hat{x}}_{4}+\varepsilon_{3}$$ (45)

$$\ddot{z}=\sum_{i=1}^{6}b_{i}{\hat{x}}_{i}+\varepsilon_{4}+{\dot{\varepsilon }}_{3}$$ (46)

$$\dddot{z}=\sum_{i=1}^{6}a_{i}{\hat{x}}_{i}+\sum_{i=1}^{6}b_{i}\varepsilon_{i}+{\dot{\varepsilon }}_{4}+{\ddot{\varepsilon }}_{3}+\frac{K_{t}G_{m}^{2}}{\left(J_{m}G_{m}^{2}+m_{r}r_{p}^{2}\right)L_{m}}u\left(t\right)=\hat{d}+bu\left(t\right)$$ (47)

The *a*_*i*_ symbols in Equation (47) are explained according to (48) to (53).


$$a_{1}=-\frac{\left(C_{m}G_{m}^{2}+C_{r}r_{p}^{2}\right)K_{c}G_{m}}{{\left(J_{m}G_{m}^{2}+m_{r}r_{p}^{2}\right)}^{2}}$$ (48)

$$a_{2}=\frac{K_{c}G_{m}}{J_{m}G_{m}^{2}+m_{r}r_{p}^{2}}$$ (49)

$$a_{3}=\frac{\left(C_{m}G_{m}^{2}+C_{r}r_{p}^{2}\right)\left(K_{c}+K_{r}r_{p}^{2}\right)}{{\left(J_{m}G_{m}^{2}+m_{r}r_{p}^{2}\right)}^{2}}$$ (50)

$$\begin{array}{c} a_{4}=-\frac{K_{c}+K_{r}r_{p}^{2}}{J_{m}G_{m}^{2}+m_{r}r_{p}^{2}}\\ +\frac{{\left(C_{m}G_{m}^{2}+C_{r}r_{p}^{2}\right)}^{2}}{{\left(J_{m}G_{m}^{2}+m_{r}r_{p}^{2}\right)}^{2}}-\frac{K_{t}^{2}G_{m}^{2}}{\left(J_{m}G_{m}^{2}+m_{r}r_{p}^{2}\right)L_{m}} \end{array}$$ (51)

$$\begin{array}{c} a_{5}=-\frac{\left(C_{m}G_{m}^{2}+C_{r}r_{p}^{2}\right)K_{t}G_{m}^{2}}{{\left(J_{m}G_{m}^{2}+m_{r}r_{p}^{2}\right)}^{2}}\\ -\frac{K_{t}R_{m}G_{m}^{2}}{\left(J_{m}G_{m}^{2}+m_{r}r_{p}^{2}\right)L_{m}} \end{array}$$ (52)

$$a_{6}=\frac{\left(C_{m}G_{m}^{2}+C_{r}r_{p}^{2}\right)G_{m}}{{\left(J_{m}G_{m}^{2}+m_{r}r_{p}^{2}\right)}^{2}}$$ (53)

Equation (54) is obtained by taking the third derivative of *e*_3_.


$${\dddot{e}}_{3}={\dddot{y}}_{1}-{\dddot{\hat{x}}}_{3}={\dddot{y}}_{1}-\dddot{z}$$ (54)

**Fig 3:**
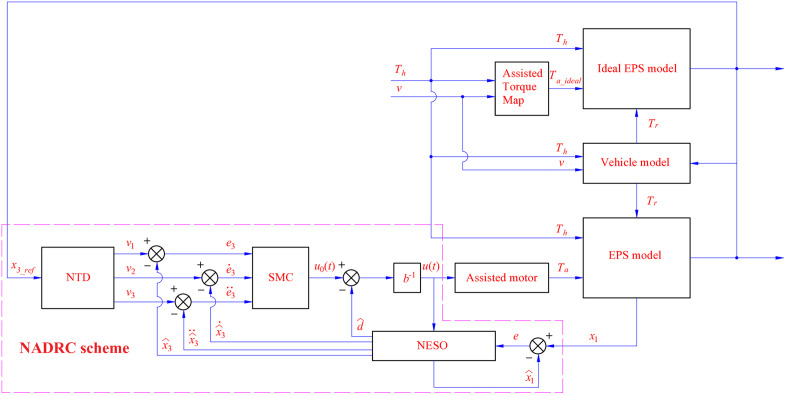
Robust control scheme.


A control law is proposed in (55), according to Nguyen and Nguyen [33] (Fig 3).

$$u\left(t\right)=\frac{u_{0}\left(t\right)-\hat{d}}{b}$$ (55)

A sliding surface (*σ*) is chosen according to (56), where *k*_*σ*_ are the coefficients chosen to ensure stability according to the Hurwitz condition.


$$\sigma ={\ddot{e}}_{3}+k_{\sigma 1}{\dot{e}}_{3}+k_{\sigma 2}e_{3}$$ (56)

Taking the derivative of the sliding surface, we get (57).


$$\dot{\sigma }={\dddot{y}}_{1}-\dddot{z}+k_{\sigma 1}{\ddot{e}}_{3}+k_{\sigma 2}{\dot{e}}_{3}$$ (57)

Initial control input (*u*_0_) is selected according to (58).


$$u_{0}\left(t\right)={\dddot{y}}_{1}+k_{\sigma 1}{\ddot{e}}_{3}+k_{\sigma 2}{\dot{e}}_{3}+k_{\sigma 3}sat\left(\sigma \right)$$ (58)

#### 2.3.1. Stability proofs.

A candidate Lyapunov function is selected according to (59), including the sliding surface (σ) representing the SMC mechanism and tracking error (*e*_*y*_) representing the NADRC mechanism.


$$\begin{array}{cc} {}*20cV\left(x\right)=\frac{\text{1}}{\text{2}}k_{y1}e_{y1}^{2}+\frac{\text{1}}{\text{2}}e_{y2}^{2}+\frac{\text{1}}{\text{2}}\sigma^{2}>0 & \forall x\neq 0 \end{array}$$ (59)

Taking the derivative of *V*(*x*) and combining it with (34), (55), (57), and (58), we get (60). The coefficients *k*_*y*2_ and *k*_*σ*3_ are chosen to be positive (as explained earlier), so (60) is negative definite. Combining (59) and (60), the system is considered stable.


$$\begin{array}{ccc} & \dot{V}\left(x\right)=k_{y1}e_{y1}{\dot{e}}_{y1}+e_{y2}{\dot{e}}_{y2}+\sigma \dot{\sigma }\\ & =k_{y1}e_{y1}e_{y2}+e_{y2}\left[-k_{y1}e_{y1}-k_{y2}sat\left(e_{y2}\right)\right]+\sigma \left({\dddot{y}}_{1}-\dddot{z}+k_{\sigma 1}{\ddot{e}}_{3}+k_{\sigma 2}{\dot{e}}_{3}\right)\\ & =-k_{y2}e_{y2}sat\left(e_{y2}\right)+\sigma \left\{{\dddot{y}}_{1}-\hat{d}-b\frac{\left[{\dddot{y}}_{1}+k_{\sigma 1}{\ddot{e}}_{3}+k_{\sigma 2}{\dot{e}}_{3}+k_{\sigma 3}sat\left(\sigma \right)\right]-\hat{d}}{b}+k_{\sigma 1}{\ddot{e}}_{3}+k_{\sigma 2}{\dot{e}}_{3}\right\}\\ & =-k_{y2}e_{y2}sat\left(e_{y2}\right)-k_{\sigma 3}\sigma sat\left(\sigma \right)<0 & \forall x\neq 0 \end{array}$$ (60)

### 2.4. ANFIS training model

The controller designed in section 2.3 generates the control input, which is used as data for a training process. In this article, we propose designing an intelligent self-learning algorithm based on the combination of fuzzy theory and neural network mechanism, called ANFIS. This algorithm comprises three inputs (simulation time, hand torque, and vehicle speed) and one output (control signal).

Let the inputs be *t*_1_, *t*_2_, and *t*_3_, and the output be *u*. Each input variable is fuzzified using seven Gaussian Membership Functions (MFs), explained as (61).


$$\begin{array}{cccc} {}*20c\mu_{A_{i}^{\left(1\right)}}\left(t_{1}\right) & \mu_{A_{j}^{\left(2\right)}}\left(t_{2}\right) & \mu_{A_{k}^{\left(3\right)}}\left(t_{3}\right) & i,j,k\in \left\{1,2,3,4,5,6,7\right\} \end{array}$$ (61)

The mathematical model of each membership function is represented by (62), where *µ*_*A*_ is degree if membership, *c* is mean of the Gaussian function and *χ* is standard deviation.


$$\mu_{A}\left(t\right)=e^{-\frac{{\left(t-c\right)}^{2}}{2\chi^{2}}}$$ (62)

The fuzzy rules are constructed according to (63), where *p*_*r*_, *q*_*r*_, and *r*_*r*_ are linear coefficients associated with inputs *t*_1_, *t*_2_, and *t*_3_, respectively, in the consequent of rule *r*. Additionally, *s*_*r*_ presents bias constant and *v*_*r*_ presents output of the consequent part of rule.


$$\begin{array}{cc} {}*20cIF & \begin{array}{ccc} {}*20ct_{1}=A_{i}^{\left(1\right)} & t_{2}=A_{j}^{\left(2\right)} & t_{3}=A_{k}^{\left(3\right)} \end{array}\\ THEN & v_{r}=p_{r}t_{1}+q_{r}t_{2}+r_{r}t_{3}+s_{r} \end{array}$$ (63)

*r* lies in the range *r* = 1, 2, 3, …, 343, and is represented through an index mapping (64).


r=(i−1)×49+(j−1)×7+k (64)

**Fig 4:**
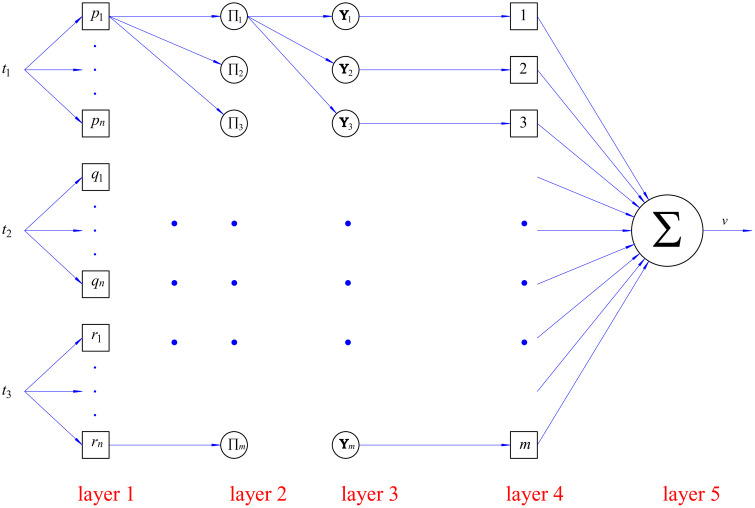
Proposed ANFIS structure.

The structure of the ANFIS algorithm (Fig 4) is constructed by five layers and is calculated as (65) to (69):

The first layer: Fuzzification


$$\begin{array}{ccc} {}*20cO_{i}^{\left(1\right)}=\mu_{A_{i}^{\left(1\right)}}\left(t_{1}\right) & O_{j}^{\left(1\right)}=\mu_{A_{j}^{\left(2\right)}}\left(t_{2}\right) & O_{k}^{\left(1\right)}=\mu_{A_{k}^{\left(3\right)}}\left(t_{3}\right) \end{array}$$ (65)

The second layer: Rule


$$w_{r}=\mu_{A_{i}^{\left(1\right)}}\left(t_{1}\right)\times \mu_{A_{j}^{\left(2\right)}}\left(t_{2}\right)\times \mu_{A_{k}^{\left(3\right)}}\left(t_{3}\right)$$ (66)

The third layer: Normalization


$${\bar{w}}_{r}=\frac{w_{r}}{\sum_{r=1}^{343}w_{r}}$$ (67)

The fourth layer: Consequent


$${\bar{w}}_{r}\times v_{r}={\bar{w}}_{r}\times \left(p_{r}t_{1}+q_{r}t_{2}+r_{r}t_{3}+s_{r}\right)$$ (68)

The final layer: Output


$$v\left(t_{1},t_{2},t_{3}\right)=\sum_{r=1}^{343}\frac{w_{r}}{\sum_{r=1}^{343}w_{r}}\times v_{r}$$ (69)

ANFIS’s hybrid learning approach combines Gradient Descent (GD) and Least Squares Estimation (LSE) to optimize the model parameters more effectively. Where each optimization strategy is applied to a particular component of the ANFIS model, this method seeks to reduce the total error by continuously changing the system’s settings. Specifically, Gradient Descent is used to maximize the parameters of the MFs in the first layer. Simultaneously, LSE modifies the consequent parameters in the rule base.

The parameters of the membership functions, such as the center *c*_*i*_ and *χ*_*i*_ of the Gaussian functions, are updated using GD. This aims to minimize the error between the predicted output and the target output, which can be introduced as (70), where *θ* represents the MF parameters, *n* is iteration, *η* is learning rate, and ∇*L*(*θ*) is the gradient of the loss function with respect to the parameters.


$$\theta^{\left(n+1\right)}=\theta^{\left(n\right)}-\eta \nabla L\left(\theta \right)$$ (70)

Once the MFs are optimized, LSE is applied to the consequent parameters of the fuzzy rules. The system can be presented in a matrix form as (71), where ***C*** is the matrix containing the data processed by the fuzzy rules, ***X*** is the vector of the consequent parameters, and ***B*** is the vector of target outputs.


C×X=B (71)

The least squares solution to this system of equations is given according to (72).


$$X={\left(C^{T}C\right)}^{-1}C^{T}B$$ (72)

This integrated methodology enables the ANFIS model to concurrently adjust the membership functions and the rule conclusions, hence increasing the precision of the fuzzy inference system. The hybrid method of learning locally optimizes membership function parameters using GD, while globally calibrating consequent parameters through LSE.

### 2.5. Training process

**Fig 5:**
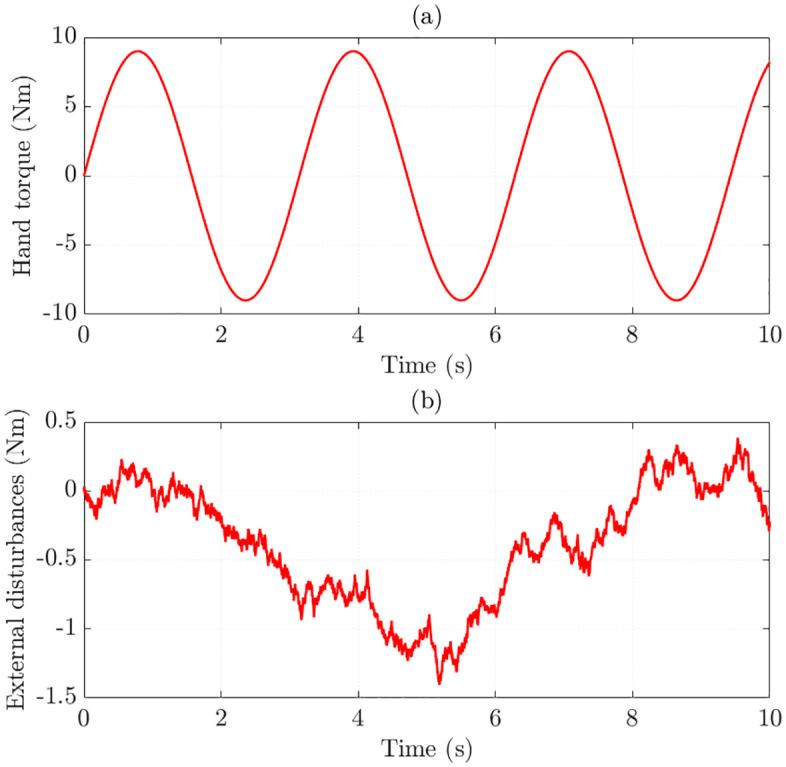
Simulation conditions. (a) Hand torque, (b) Disturbances.

Once the algorithm design is completed, the training process will be carried out. The proposed algorithm trains a large dataset with three inputs and one output. The first input is the simulation time, which is set to 10 seconds with varying time steps. The second input is the hand torque, which changes over time and is illustrated in Fig 5a. The influence of external disturbances, caused by a white noise source, is shown in Fig 5b. The vehicle speed (the third input) varies across several cases, including *v* = 20 km/h, 30 km/h, 40 km/h, 50 km/h, 60 km/h, and 70 km/h. The output is the control input (voltage signal), designed according to Equation (55).

The steering input is designed as a sinusoidal hand torque profile, as illustrated in Fig 5a. This profile is selected to excite the EPS system over a wide range of operating conditions, allowing the proposed controller to be evaluated under continuously varying torque demands. The sinusoidal profile allows the torque to alternate repeatedly between positive and negative values, which helps assess the controller’s capability in controlling steering efforts in both directions and responding to variations in load. The torque amplitude is fixed at ±10 Nm, and the chosen period produces several oscillations within the simulation time, ensuring that the dataset contains sufficient variation for practical training and validation.

The training dataset is derived from previous simulation results, consisting of 25972 scenarios corresponding to the abovementioned conditions. The grid partition structure is based on 7 Gaussian Membership Functions (MFs), and the hybrid method is selected as the optimal calculation approach. The training process is carried out over 250 epochs.

A sensitivity analysis is conducted to justify the selection of the primary hyperparameters in the proposed ANFIS model, namely the number of Gaussian MFs per input and the number of training epochs. The analysis is performed by varying one parameter at a time while keeping all others fixed, and evaluating the Root Mean Square Error (RMSE) on the testing dataset. The results indicated that the chosen configuration of 7 Gaussian MFs per input provided an appropriate balance between model complexity and prediction accuracy. Similarly, the training process showed that increasing the number of epochs improved convergence up to 250 epochs, beyond which no significant accuracy gain was observed. Increasing the number of MFs or epochs further would lead to longer computation times and higher computational resource consumption, yielding only marginal accuracy improvements. Conversely, reducing these values would shorten the computation time but result in a considerable degradation in prediction quality. These findings confirm that the proposed configuration (7 Gaussian MFs and 250 training epochs) provides high accuracy and computational efficiency for the given dataset.

**Fig 6:**
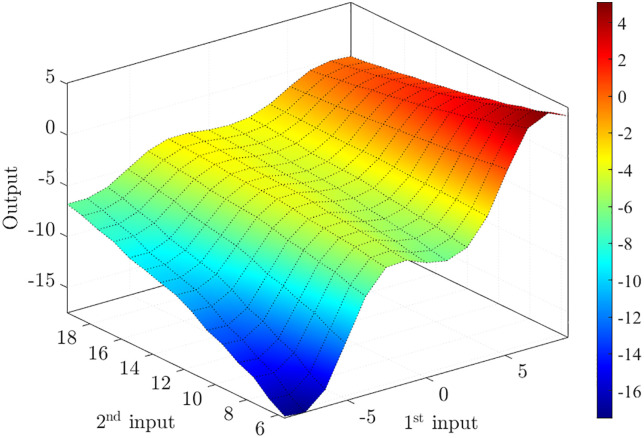
Fuzzy surface.

The training results indicate that the minimum RMSE, also referred to as the average testing error, is 0.116861. Based on fuzzy rules, the fuzzy surface is illustrated in Fig 6 with two inputs and one output. The third input is represented through the connections in the fuzzy surface.

## 3. Validation and discussion

### 3.1. Simulation conditions

In this article, the performance of the proposed controller is investigated in three cases, corresponding to three different speeds (*v*_1_ = 20 km/h, *v*_2_ = 55 km/h, and *v*_3_ = 73 km/h). The primary purpose of this is to evaluate the data training performance of the proposed algorithm, compared to other methods. The system specifications are referred to in [33].

The simulation results include control input, state variables, and motor current. The results obtained from the proposed ANFIS mechanism (ANFIS_1_) are compared with those of another ANFIS mechanism (ANFIS_2_) and the BPNN mechanism. The training characteristics of the above algorithms are listed in Table 1 below.

**Table 1:** Characteristics of training algorithms.

Algorithms	Characteristics	Training speed
ANFIS_1_	It has a 5-layer structure with 7 Membership Functions (MFs) based on Gaussian functions. The training process takes place over 250 epochs.	Slow
ANFIS_2_	It has a 5-layer structure with 6 Membership Functions (MFs) based on Gaussian functions. The training process takes place over 150 epochs.	Average
BPNN	It has a 2-layer hidden structure (the first layer consists of 25 neurons, while the second layer contains 20 neurons). The training process takes place over 1000 epochs.	Fast

### 3.2. Simulation results

#### 3.2.1. *v*_1_ = 20 km/h.

**Fig 7:**
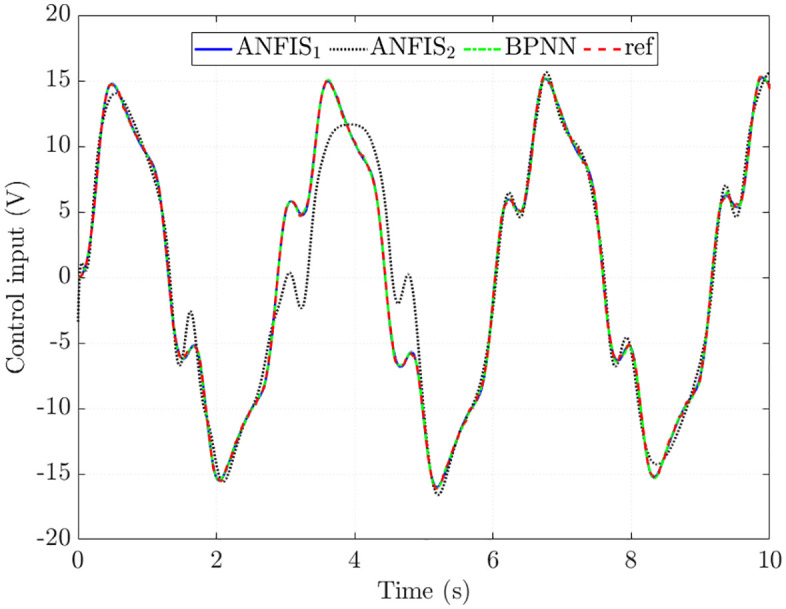
Control input in the first case.

The training results in the first case (*v*_1_ = 20 km/h) are shown in Fig 7. Generally, the trained signal obtained from ANFIS_1_ and BPNN algorithms closely follows the reference signal with minor errors. The Root Mean Square Error (RMSE) in the control input of ANFIS_1_ is 1.256%, slightly higher than that of BPNN (0.887%). In contrast, the tracking error of ANFIS_2_ is more significant at some points, causing the RMSE to increase to 21.428%. The Integral Absolute Error (IAE) values of ANFIS_1_, ANFIS_2_, and BPNN are 1.118%, 14.410%, and 0.768%, respectively. It can be seen that the training accuracy of ANFIS_1_ and BPNN algorithms is exceptionally high.

**Fig 8:**
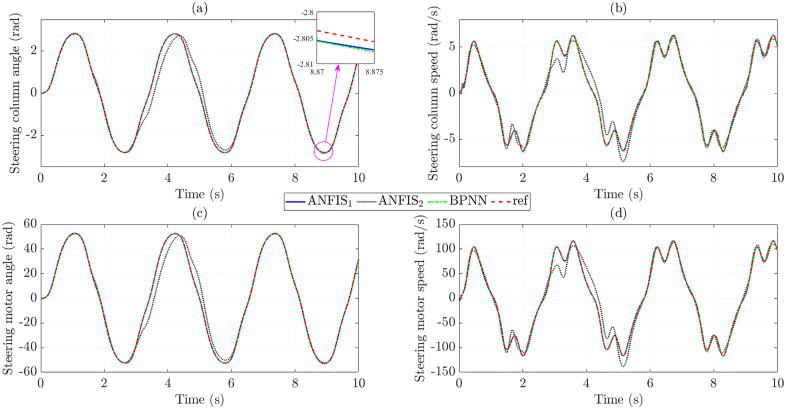
State variables in the first case. (a) Steering column angle signals, (b) Steering column speed signals, (c) Steering motor angle signals, (d) Steering motor speed signals.

Fig 8 illustrates the changes in the state variables when the trained control input controls the system. The calculated results show that the RMSE in steering column angle obtained from ANFIS_1_ and BPNN is extremely small, only 0.108% and 0.064%, respectively, while the data obtained from ANFIS_2_ is much larger (Fig 8a). Taking the derivative of the first state variable (*x*_1_), we obtain the steering column speed (*x*_2_) as shown in Fig 8b. Although the RMSE in steering column speed is larger than that in steering column angle, these values are still minimal (1.614% for ANFIS_1_, 17.732% for ANFIS_2_, and 1.154% for BPNN), which proves the stability of the trained data. The changes in steering motor angle (Fig 8c) and steering motor speed (Fig 8d) are not much different from those of steering column angle and steering column speed, respectively.

**Fig 9:**
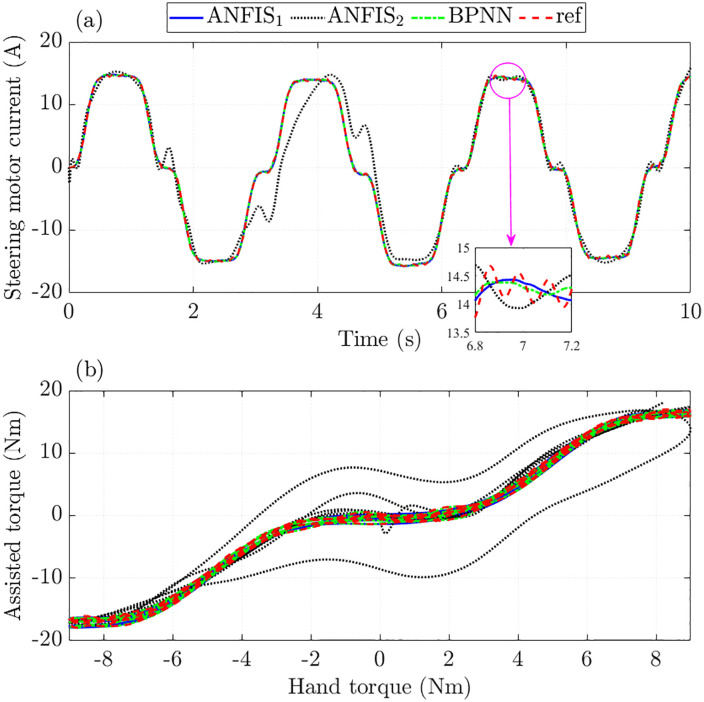
Assisted performance in the first case. (a) Steering motor current signals, (b) Assisted and hand torque.

The steering performance is evaluated through the motor current signal and assisted torque. The simulation results in Fig 9a show that the steering motor current obtained from ANFIS_1_ and BPNN algorithms closely follows the reference value with RMSE not exceeding 1.6%. Looking at the window plot in Fig 9a more clearly, it can be seen that the trained signal tends to move smoothly than the original reference signal.

The curves in Fig 9b illustrate the dependence of assisted torque on hand torque. In general, the tracking error of ANFIS_2_ is quite large, which is caused by insufficient training (small number of MFs and epochs). In contrast, the signals obtained from the training process by ANFIS1 and BPNN show superior tracking ability under this investigated condition.

The simulation results for the first case (*v*_1_ = 20 km/h) are listed in Table 2. It should be noted that these figures have been rounded.

**Table 2:** Training errors in the first case (%).

Criterias	RMSE			IAE		
Training algorithms	ANFIS_1_	ANFIS_2_	BPNN	ANFIS_1_	ANFIS_2_	BPNN
Steering column angle	0.108	14.499	0.064	0.098	8.847	0.056
Steering column speed	1.614	17.732	1.154	1.491	14.005	1.042
Steering motor angle	0.084	14.781	0.045	0.075	8.989	0.041
Steering motor speed	0.649	17.707	0.346	0.598	13.652	0.307
Steering motor current	1.596	23.308	1.170	1.517	16.807	1.080
Control input	1.256	21.428	0.887	1.118	14.410	0.768

The simulation results in the first case show that ANFIS_1_ and BPNN algorithms provide superior performance in training data, while the performance of ANFIS_2_ is significantly worse. These are the results obtained in the case of the vehicle moving at a speed of *v*_1_ = 20 km/h, which is the case of training with complete and accurate data. It is necessary to investigate the performance of the training algorithms at different speeds (non-trained data) to evaluate the performance of the training algorithm comprehensively.

#### 3.2.2. *v*_2_ = 55 km/h.

In the second case, the vehicle speed is increased to 55 km/h. It is worth noting that the training data only includes speeds *v* = 20, 30, 40, 50, 60, and 70 km/h. The speed investigated in this case (*v*_2_ = 55 km/h) is within the training range but not the trained value. Therefore, the investigation at *v*_2_ offers significant potential for evaluating the performance of training algorithms.

**Fig 10:**
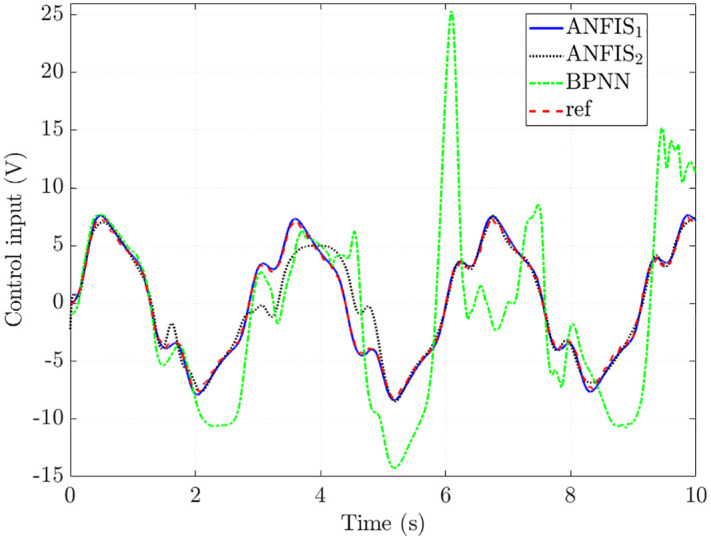
Control input in the second case.

The simulation results in Fig 10 show that the control input obtained from the ANFIS1 algorithm closely follows the reference value with minor errors (RMSE = 4.542% and IAE = 4.188%). The results obtained from ANFIS_2_ tend to follow the reference signal, but its error is larger than that of ANFIS_1_. This is caused by the change in the algorithm structure (reducing the number of MFs and epochs), causing the training error to increase to 27.371% (RMSE) and 17.415% (IAE). The interpolation ability of BPNN algorithms is low, causing the training error to increase to 114.051% (RMSE) and 91.095% (IAE). The main reason for this is the overfitting phenomenon in training, causing the BPNN algorithm to lose the ability to interpolate in other conditions.

**Fig 11:**
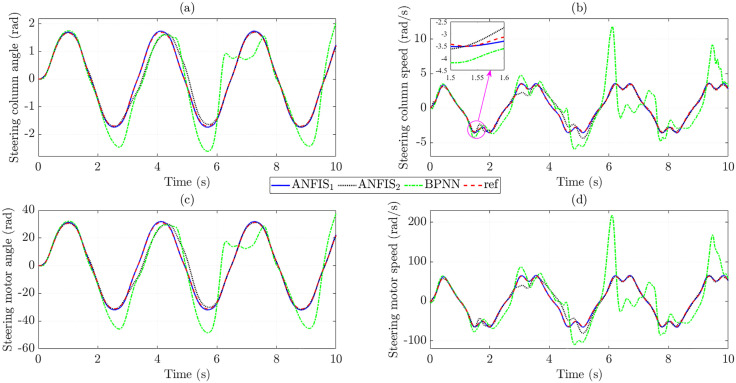
State variables in the second case. (a) Steering column angle signals, (b) Steering column speed signals, (c) Steering motor angle signals, (d) Steering motor speed signals.

The increase in the control input’s tracking error causes the state variables’ tracking error to increase. The simulation results in Fig 11 show that the training error obtained from the BPNN algorithm is significant, much larger than that of the proposed ANFIS. In contrast, the accuracy of the results of the ANFIS1 algorithm is remarkably high (up to more than 95%), while the accuracy of ANFIS_2_ is only greater than 80%.

**Fig 12:**
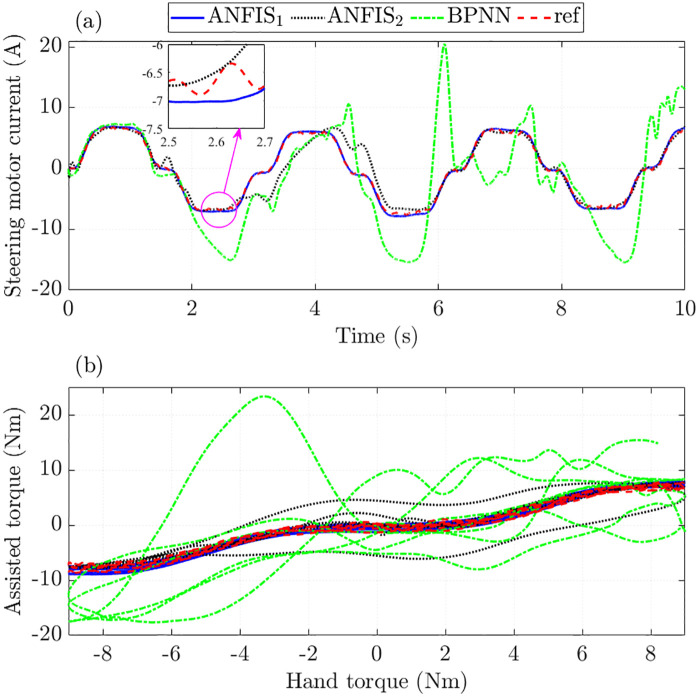
Assisted performance in the second case. (a) Steering motor current signals, (b) Assisted and hand torque.

The simulation results in Fig 12a show that the steering motor current signal obtained from ANFIS_1_ closely follows the reference signal with RMSE and IAE of 6.054% and 5.941%, respectively, much lower than that of ANFIS_2_ (33.717% and 23.596%, respectively). The control performance of the system drops sharply once the BPNN mechanism is used to train the control input, causing the assisted torque not to follow the proposed initial rule (Fig 12b).

In conclusion, ANFIS algorithms still maintain good training ability with minor errors once the MFs and epochs are appropriately selected. This is ensured through nonlinear interpolation for untrained cases. In contrast, the overfitting phenomenon of the BPNN algorithm causes the interpolation performance to deteriorate sharply, leading to increased errors in various investigated cases.

The simulation data for the second case are listed in Table 3 below.

**Table 3:** Training errors in the second case (%).

Criterias	RMSE			IAE		
Training algorithms	ANFIS_1_	ANFIS_2_	BPNN	ANFIS_1_	ANFIS_2_	BPNN
Steering column angle	2.258	14.858	45.589	2.224	8.729	39.280
Steering column speed	3.476	18.222	86.888	3.174	13.672	67.761
Steering motor angle	2.338	15.387	47.064	2.305	9.025	40.618
Steering motor speed	2.528	18.440	88.574	2.458	13.440	69.289
Steering motor current	6.054	33.717	116.732	5.941	23.596	102.424
Control input	4.542	27.371	114.051	4.188	17.415	91.095

#### 3.2.3. *v*_3_ = 73 km/h.

In the second case (*v*_2_ = 55 km/h), the training algorithms used nonlinear interpolation to determine values within the training range. An extended investigation is performed on the third case (*v*_3_ = 73 km/h), which has velocity values that fell outside the training range. This will force the training algorithms to use prediction (extrapolation) to find the required data.

**Fig 13:**
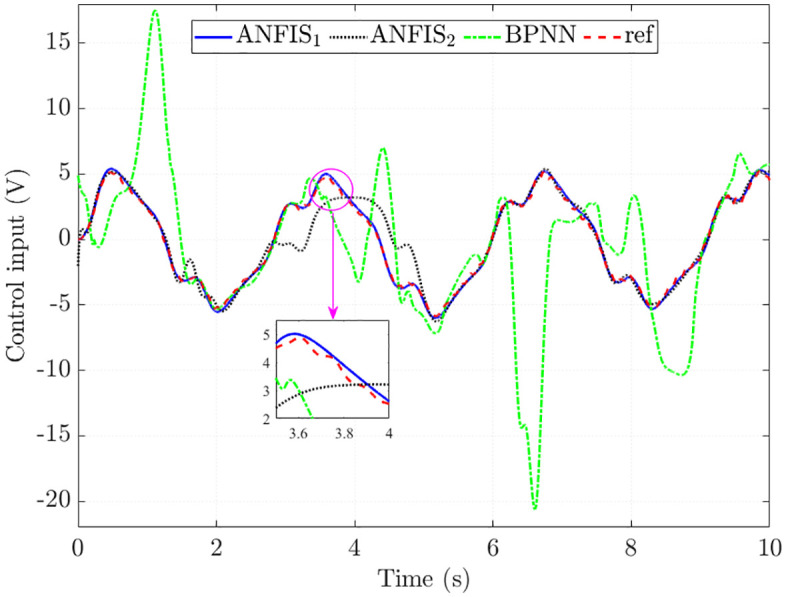
Control input in the third case.

Fig 13 shows the time variation in the trained signals. The tracking error of the BPNN algorithm is significant, while the RMSE and IAE of ANFIS_1_ are only 6.122% and 5.630%. If the algorithm structure is not chosen correctly (ANFIS_2_), these values can increase to 32.707% and 21.321%, which are still much lower than BPNN. Looking at the window plot in Fig 13 more closely, it is clear that the ANFIS_1_ signal tends to move smoothly instead of being zigzag like the reference signal. This shows that the ANFIS algorithm can generalize to the training data.

**Fig 14:**
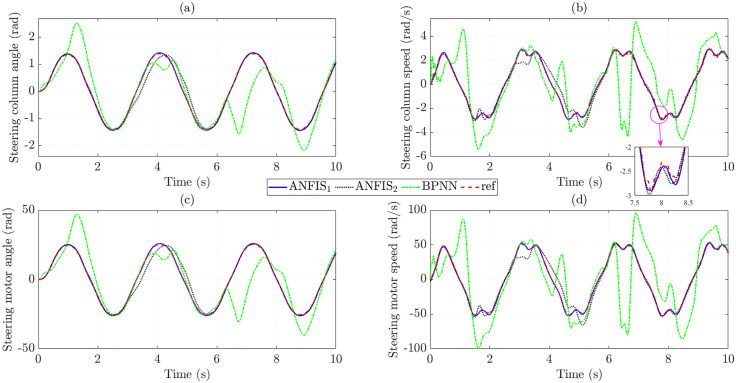
State variables in the third case. (a) Steering column angle signals, (b) Steering column speed signals, (c) Steering motor angle signals, (d) Steering motor speed signals.

Significant control input errors lead to increased state variables tracking errors (Fig 14). This occurs when the BPNN algorithm is used to train the data. In contrast, the ANFIS algorithm maintains stability in training the data, even when the investigated conditions change (actual speed is outside the training range).

**Fig 15:**
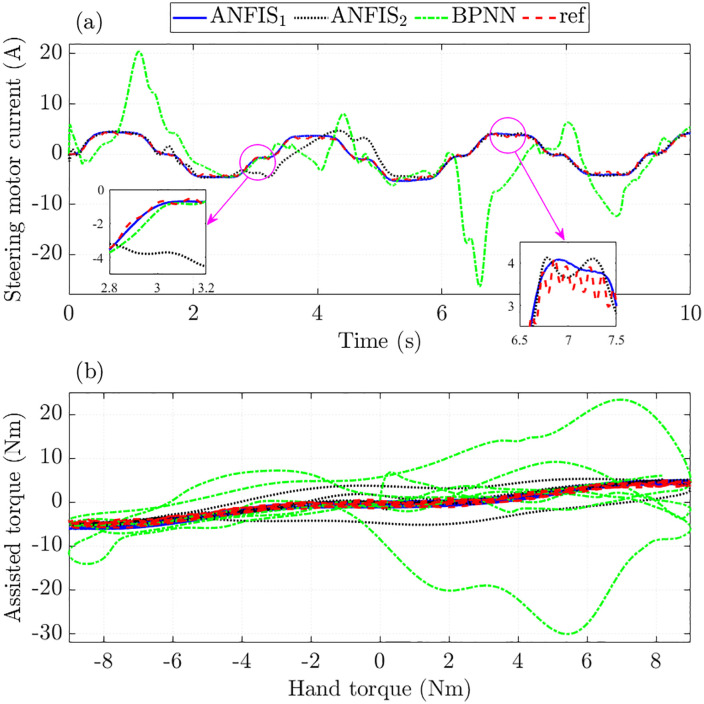
Assisted performance in the third case. (a) Steering motor current signals, (b) Assisted and hand torque.

The steering motor current obtained from the ANFIS_1_ algorithm closely follows the reference signal with RMSE and IAE not exceeding 10% (Fig 15a). This shows the superior ability to predict (extrapolate) the output results of the proposed ANFIS. If the training algorithm is designed with appropriate MFs and epochs, the accuracy of the results can be significantly improved.

The calculation results obtained in the final investigated case are listed in Table 4.

**Table 4:** Training errors in the second case (%).

Criterias	RMSE			IAE		
Training algorithms	ANFIS_1_	ANFIS_2_	BPNN	ANFIS_1_	ANFIS_2_	BPNN
Steering column angle	2.566	15.229	69.080	2.513	9.637	49.796
Steering column speed	4.117	18.902	101.403	3.711	13.988	83.119
Steering motor angle	2.680	15.919	72.141	2.630	10.082	52.053
Steering motor speed	2.906	19.259	104.720	2.783	13.753	86.055
Steering motor current	9.275	45.517	213.746	9.021	32.388	156.693
Control input	6.122	32.707	166.090	5.630	21.321	107.409

### 3.3. Discussion

Overall, the ANFIS algorithm demonstrates a notable advantage in handling training data compared to the conventional BPNN. The validation error remains minimal for the training dataset, not exceeding 1.7%. In the case of interpolated data (i.e., data within the training domain), the maximum observed error is approximately 6%. For extrapolated data (i.e., data outside the training domain), the training error remains under 9.3%. Furthermore, the proposed ANFIS-based approach effectively mitigates overfitting, a standard limitation in traditional ANN models. Notably, the intelligent self-learning mechanism in the proposed model enables it to generalize nonlinear patterns across varying operating conditions.

It is important to emphasize that the performance of the training process is highly dependent on the algorithm’s configuration. An insufficient number of membership functions (MFs) and training epochs can lead to significant errors in the resulting model. Conversely, excessive MFs and epochs increase computational complexity and resource consumption, prolonging training time. Additionally, overfitting may occur in such cases, which can adversely affect the interpolation accuracy and the model’s predictive capability.

To the best of the authors’ knowledge, to date, only the present study and the work presented in [40] have employed an ANFIS-based scheme for EPS control, whereas [47] demonstrates an application of ANFIS solely for optimizing steering control in a steer-by-wire system. The ANFIS control framework proposed herein exhibits a higher level of structural complexity than that in [40], and it is trained with a substantially larger dataset. Consequently, the accuracy of the obtained results is markedly enhanced. Furthermore, compared with other ANFIS-based controllers reported in [43,44,48,49], the proposed approach is more advanced in terms of scale and architecture.

## 4. Conclusion

This article presented the design of an intelligent self-learning algorithm based on integrating neural networks and fuzzy logic systems (ANFIS) to enhance training performance on large-scale datasets. The training data were derived from a controller used in the EPS system. The training process was carried out on a high-performance PC, and the results were compared with those of other algorithms to validate the effectiveness of the proposed ANFIS approach.

The findings indicate that the training error of the proposed algorithm is negligible when the test inputs match the training data. In addition, the errors for interpolated (within range) and extrapolated (out of range) inputs remain relatively low. The proposed ANFIS structure also helps eliminate the typical problems of overfitting and underfitting, which are often encountered in conventional BPNN models. Maintaining low control input error contributes to reducing output state variable errors under different operating conditions.

The proposed intelligent self-learning algorithm shows strong potential for training large datasets under varying conditions. However, several drawbacks remain, including: the first, extended training time; the second, high computational resource demands; and the third, noticeable prediction error for data outside the training range. Finally, determining an optimal algorithm structure with the most suitable parameters remains challenging. These issues are expected to be addressed in future research.

## References

[1] Xia L, Jiang H. An electronically controlled hydraulic power steering system for heavy vehicles. Adv Mech Eng. 2016;8(11). doi: 10.1177/1687814016679566

[2] Baharom MB, Hussain K, Day AJ. Design of full electric power steering with enhanced performance over that of hydraulic power-assisted steering. Proc Inst Mech Eng, Part D: J Automobile Eng. 2013;227(3):390–9. doi: 10.1177/0954407012468413

[3] Turan A. Improved PID control design for electric power steering DC motor. IEEE Access. 2025;13:6080–8. doi: 10.1109/access.2024.3524303

[4] Hanifah RA, Toha SF, Ahmad S, Hassan MOHDK. Swarm-intelligence tuned current reduction for power-assisted steering control in electric vehicles. IEEE Trans Ind Electron. 2018;65(9):7202–10. doi: 10.1109/tie.2017.2784344

[5] Zheng Z, Wei J. Research on electric power steering fuzzy PI control strategy based on phase compensation. Int J Dynam Control. 2022;11(4):1867–79. doi: 10.1007/s40435-022-01077-2

[6] Dai C, Chen G, Zong C, Zhang B. Precise compound control of loading force for electric load simulator of electric power steering test bench. Chin J Mech Eng. 2022;35(1). doi: 10.1186/s10033-021-00670-4

[7] Li Y, Wu G, Wu L, Chen S. Electric power steering nonlinear problem based on proportional–integral–derivative parameter self-tuning of back propagation neural network. Proc Inst Mech Eng Part C: J Mech Eng Sci. 2020;234(23):4725–36. doi: 10.1177/0954406220926549

[8] Mehrabi N, McPhee J, Azad NL. Design and evaluation of an observer-based disturbance rejection controller for electric power steering systems. Proc Inst Mech Eng Part D: J Automobile Eng. 2015;230(7):867–84. doi: 10.1177/0954407015596275

[9] Liu X, Pang H, Shang Y, Wu W. Optimal design of fault‐tolerant controller for an electric power steering system with sensor failures using genetic algorithm. Shock Vibr. 2018;2018(1). doi: 10.1155/2018/1801589

[10] Nguyen TA. A linear quadratic tracking control strategy based on fuzzy for electric power steering systems. Adv Mech Eng. 2025;17(2). doi: 10.1177/16878132251323407

[11] Yamamoto K, Sename O, Koenig D, Moulaire P. Design and experimentation of an LPV extended state feedback control on Electric Power Steering systems. Control Eng Pract. 2019;90:123–32. doi: 10.1016/j.conengprac.2019.06.004

[12] Zhao W, Zhang H. Coupling control strategy of force and displacement for electric differential power steering system of electric vehicle with motorized wheels. IEEE Trans Veh Technol. 2018;67(9):8118–28. doi: 10.1109/tvt.2018.2850154

[13] Zhao W, Li Y, Wang C, Zhao T, Gu X. H∞ control of novel active steering integrated with electric power steering function. J Cent South Univ. 2013;20(8):2151–7. doi: 10.1007/s11771-013-1719-0

[14] Lee D, Kim K-S, Kim S. Controller design of an electric power steering system. IEEE Trans Contr Syst Technol. 2018;26(2):748–55. doi: 10.1109/tcst.2017.2679062

[15] Nguyen DN, Nguyen TA. Proposing a BSPID control strategy considering external disturbances for electric power steering (EPS) systems. IEEE Access. 2023;11:143230–49. doi: 10.1109/access.2023.3343914

[16] Nguyen TA, Iqbal J. Genetic algorithm inspired optimal integrated nonlinear control technique for an electric power steering system. J Braz Soc Mech Sci Eng. 2024;46(11). doi: 10.1007/s40430-024-05255-5

[17] Nguyen DN, Nguyen TA. Fuzzy backstepping control to enhance electric power steering system performance. IEEE Access. 2024;12:88681–95. doi: 10.1109/access.2024.3419001

[18] Nguyen TA. Development of a novel integrated control strategy for automotive electric power steering systems. IEEE Trans Automat Sci Eng. 2025;22:926–43. doi: 10.1109/tase.2024.3356509

[19] Marouf A, Djemai M, Sentouh C, Pudlo P. A new control strategy of an electric-power-assisted steering system. IEEE Trans Veh Technol. 2012;61(8):3574–89. doi: 10.1109/tvt.2012.2209689

[20] Khasawneh L, Das M. A robust electric power-steering-angle controller for autonomous vehicles with disturbance rejection. Electronics. 2022;11(9):1337. doi: 10.3390/electronics11091337

[21] Kim S, Choi M, Sung J. Experimental verifications of electric power steering controller based on discrete-time sliding mode control with disturbance observer. IntJ Automot Technol. 2023;24(3):883–8. doi: 10.1007/s12239-023-0072-z

[22] Li Y, Shim T, Wang D, Offerle T. Model reference control-based steering feel improvement for electric power-assisted steering system. J Dynam Syst Measure Control. 2023;145(4). doi: 10.1115/1.4056532

[23] Kim G, You S, Lee S, Shin D, Kim W. Robust nonlinear torque control using steering wheel torque model for electric power steering system. IEEE Trans Veh Technol. 2023;72(7):9555–60. doi: 10.1109/tvt.2023.3248301

[24] Lee D, Yi K, Chang S, Lee B, Jang B. Robust steering-assist torque control of electric-power-assisted-steering systems for target steering wheel torque tracking. Mechatronics. 2018;49:157–67. doi: 10.1016/j.mechatronics.2017.12.007

[25] Nguyen TA, Iqbal J. Improving stability and adaptability of automotive electric steering systems based on a novel optimal integrated algorithm. Eng Comput. 2024;41(4):991–1034. doi: 10.1108/ec-10-2023-0675

[26] Anh Nguyen T. A new approach to an optimal integrated control strategy for electric steering systems. Ain Shams Eng J. 2024;15(8):102881. doi: 10.1016/j.asej.2024.102881

[27] Lu S, Lian M, Liu M, Cho C, Piao C. Adaptive fuzzy sliding mode control for electric power steering system. J Mech Sci Technol. 2017;31(6):2643–50. doi: 10.1007/s12206-017-0507-4

[28] Nguyen TA. Fuzzy sliding mode control based on an adaptive sliding surface and extended state observer for automotive electric power steering. Adv Mech Eng. 2025;17(1). doi: 10.1177/16878132251315517

[29] Nguyen TA. Proposing a novel nonlinear integrated control technique for an electric power steering system to improve automotive dynamic stability. Proc Inst Mech Eng Part K: J Multi-body Dynam. 2024;238(3):445–60. doi: 10.1177/14644193241267200

[30] Nguyen TA. Designing a new integrated control solution for electric power steering systems based on a combination of nonlinear techniques. PLoS One. 2024;19(9):e0308530. doi: 10.1371/journal.pone.0308530 39283927 PMC11404794

[31] Na S, Li Z, Qiu F, Zhang C. Torque control of electric power steering systems based on improved active disturbance rejection control. Math Problem Eng. 2020;2020:1–13. doi: 10.1155/2020/6509607

[32] Zheng Z, Wei J. Research on active disturbance rejection control strategy of electric power steering system under extreme working conditions. Measure Control. 2023;57(1):90–100. doi: 10.1177/00202940231192986

[33] Nguyen TA, Nguyen TL. Nonlinear active disturbance rejection mechanism based sliding mode control for enhancing electric power assisted steering performance. PLoS One. 2025;20(4):e0321664. doi: 10.1371/journal.pone.0321664 40215223 PMC11990780

[34] Alabe LW, Kea K, Han Y, Min YJ, Kim T. A deep learning approach to detect anomalies in an electric power steering system. Sensors (Basel). 2022;22(22):8981. doi: 10.3390/s22228981 36433579 PMC9699008

[35] Hartono R, Rok Cha H, Shin KJ. Design of electric power steering system identification and control for autonomous vehicles based on artificial neural network. IEEE Access. 2024;12:108460–71. doi: 10.1109/access.2024.3387460

[36] Amirkhani A, Shirzadeh M, Heydari J. Automotive electric power steering control with robust observer based neuroadaptive type-2 radial basis function methodology. IEEE Open J Veh Technol. 2024;5:592–605. doi: 10.1109/ojvt.2024.3383516

[37] You S, Kim G, Lee S, Shin D, Kim W. Neural approximation-based adaptive control using reinforced gain for steering wheel torque tracking of electric power steering system. IEEE Trans Syst Man Cybern, Syst. 2023;53(7):4216–25. doi: 10.1109/tsmc.2023.3241452

[38] Lin F-J, Chen S-G, Sun I-F. Intelligent sliding-mode position control using recurrent wavelet fuzzy neural network for electrical power steering system. Int J Fuzzy Syst. 2017;19(5):1344–61. doi: 10.1007/s40815-017-0342-x

[39] Hung Y-C, Lin F-J, Hwang J-C, Chang J-K, Ruan K-C. Wavelet fuzzy neural network with asymmetric membership function controller for electric power steering system via improved differential evolution. IEEE Trans Power Electron. 2015;30(4):2350–62. doi: 10.1109/tpel.2014.2327693

[40] Ramos-Fernández JC, López-Morales V, Márquez-Vera MA, Pérez JMX, Suarez-Cansino J. Neuro-fuzzy modelling and stable pd controller for angular position in steering systems. IntJ Automot Technol. 2021;22(6):1495–503. doi: 10.1007/s12239-021-0129-9

[41] Sharma SK, Sharma RC, Choi Y, Lee J. Modelling and dynamic analysis of adaptive neuro-fuzzy inference system-based intelligent control suspension system for passenger rail vehicles using magnetorheological damper for improving ride index. Sustainability. 2023;15(16):12529. doi: 10.3390/su151612529

[42] Nitish N, Singh AK. Design of enhanced active suspension system with metaheuristic-based LQG control and ANFIS-based actuation. Mech Based Design Struct Mach. 2025;53(9):6196–227. doi: 10.1080/15397734.2025.2480442

[43] Nguyen TA. Proposing a new control method for active stabilizer bars using an intelligent self-learning algorithm. Proc Inst Mech Eng Part D: J Automobile Eng. 2024;239(8):3489–510. doi: 10.1177/09544070241247992

[44] Nguyen TA. Adaptive neuro-fuzzy inference control for active stabilizer bars based on multiple data sources. Sci Prog. 2024;107(3). doi: 10.1177/00368504241274976 39252502 PMC11394359

[45] Suhail M, Akhtar I, Kirmani S, Jameel M. Development of progressive fuzzy logic and ANFIS control for energy management of plug-in hybrid electric vehicle. IEEE Access. 2021;9:62219–31. doi: 10.1109/access.2021.3073862

[46] Gómez-Barroso Á, Alonso Tejeda A, Vicente Makazaga I, Zulueta Guerrero E, Lopez-Guede JM. Dynamic programming-based ANFIS energy management system for fuel cell hybrid electric vehicles. Sustainability. 2024;16(19):8710. doi: 10.3390/su16198710

[47] Ali M, Muhlasin, Nurohmah H, Raikhani A, Sopian H, Sutantra N. Combined ANFIS method with FA, PSO, and ICA as Steering Control Optimization on Electric Car. Proceedings of the Electrical Power, Electronics, Communications, Controls and Informatics Seminar (EECCIS). 2018. pp. 299–304. doi: 10.1109/EECCIS.2018.8692885

[48] Senthil Kumar P, Sivakumar K, Kanagarajan R, Kuberan S. Adaptive Neuro Fuzzy Inference System control of active suspension system with actuator dynamics. J Vibroeng. 2018;20(1):541–9. doi: 10.21595/jve.2017.18379

[49] Nugroho PW, Li W, Du H, Alici G, Yang J. An adaptive neuro fuzzy hybrid control strategy for a semiactive suspension with magneto rheological damper. Adv Mech Eng. 2014;6:487312. doi: 10.1155/2014/487312

